# Analysis of Kinship and Population Genetic Structure of 53 Apricot Resources Based on Whole Genome Resequencing

**DOI:** 10.3390/cimb46120844

**Published:** 2024-12-13

**Authors:** Qirui Xin, Jun Qing, Yanhong He

**Affiliations:** College of Forestry, Inner Mongolia Agricultural University, Hohhot 010019, China; xinqr804803416@163.com (Q.X.); qingjun8103@163.com (J.Q.)

**Keywords:** common apricot, whole genome resequencing, SNP, population genetic structure

## Abstract

Based on the single nucleotide polymorphism (SNP) markers developed by whole genome resequencing (WGRS), the relationship and population genetic structure of 53 common apricot (*P. armeniaca*) varieties were analyzed to provide a theoretical basis for revealing the phylogenetic relationship and classification of the common apricot. WGRS was performed on 53 common apricot varieties, and high-quality SNP sites were obtained after alignment with the “*Yinxiangbai*” apricot genome as a reference. Phylogenetic analysis, G matrix analysis, principal component analysis, and population structure analysis were performed using Genome-wide Complex Trait Analysis (GCTA), FastTree, Admixture, and other software. The average comparison ratio between the sequencing results and the reference genome was 97.66%. After strict screening, 88,332,238 high-quality SNP sites were finally obtained. Based on the statistical SNP variation type, it was found that *LNLJX* had the largest number of variations (3,951,322) and the lowest base transition/base transversion ratio (ts/tv = 1.77), indicating that its gene exchange events occurred less frequently. Based on the SNP point estimation of the relationship and genetic distance between samples, the relationship between species was 1.41–0.01, among which *PLDJX* and *BK1* had the closest relationship of 1.41, and *YZH* and *LGWSX* had the farthest relationship of 0.01. The genetic distance between species was 0.00367–0.264344, the genetic distance between *HMX* and *JM* was the closest, and the genetic distance between *WYX* and *YX* was the farthest, which was the largest. Phylogenetic tree, PCA, and genetic structure analysis results all divided 53 common apricot varieties into four groups, and the classification results were consistent. The SNP markers mined using WGRS technology are useful not only to analyze the variation of common apricots, but also to effectively identify their kinship and genetic structure, which plays a critical role in the classification and utilization of common apricot germplasm resources.

## 1. Introduction

Apricot (*Armeniaca vulgaris* Lam.) belongs to the *Armeniaca* genus plant of the Rosaceae (Rosaceae) and is a diploid temperate deciduous tree (2*n* = 16) with a genome size of about 245.07~291.59 Mb [1]. There are 10 species of apricot in the world. Among them, the most widely planted, the oldest, and the most diversified is the common apricot (*P. armeniaca*), which has a history spanning more than 3000 years. Owing to its long history and frequent germplasm exchange between taxa, it is also one of the least understood fruiting resources [2,3]. Due to their strong adaptability, resistance to light, cold, drought, and sand, as well as their high economic benefit, apricots are widely planted in the “three near-north regions” of China. It is important that common apricot relatives can be distinguished.

The study of the genetic diversity and genetic structure of species can not only reveal the level of genetic variation, spatial and temporal distribution, and the species’ relationship with the surrounding ecosystem, but can also aid analysis of a species’ evolutionary history and evolutionary potential, providing important information for their future development and a scientific basis for species protection and resource development and utilization [4]. By using SSR molecular markers and fluorescent capillary electrophoresis detection technology, the genetic diversity, kinship, and genetic structure of common apricot germplasm resources were analyzed, and the kinship and genetic background of some important apricot germplasm resources were clarified [5]. Yang et al. found that Limeixing has closer convergence with Li by analyzing the PCR amplification of the RAPD and S alleles of Limei apricot and other *Prunus salicina* and *Prunus mume* varieties around the world [6]. Qiuping Zhang investigated the genetic diversity and population structure of 67 north China ecological groups of ordinary apricots using SSR technology. It was found that when K = 4, except the kernel apricot, the north China ecological group of common apricots can be divided into three subgroups. Moreover, the north China ecological group of ordinary apricots has rich genetic diversity, while the kernel apricot has a narrow genetic basis, but has more unique allelic variation and a unique blood relationship, The same geographical origin is not necessarily observed for the same group [7]. 

With the rapid development of sequencing technology and sequencing cost reduction, SNP markers are being increasingly widely applied to plant population genetics research. Based on WGRS, individual and population genetic variation site datasets can be analyzed to understand the differences between individuals and groups. This technique has been widely used in trait gene positioning, genetic map construction, origin evolution, and other aspects of genetic study. Revealing genetic diversity and kinship is essential for conserving germplasm resources, improving breeding efficiency, and enhancing competitiveness. Accurate assessment of relatives and genealogical records is critical to avoid inbreeding, maintain genetic diversity, improve species adaptability and viability, guide breeding decisions, ensure the speed and quality of new breed development, and ensure the stability and economic efficiency of agricultural output. The small size and simple structure of the genomes of fruit trees such as peach [8], plum [9], and cherry [10] trees have attracted the attention of researchers. The first high-quality genome of apricot was released in 2019, with a size of 221.9 Mb, including an overlapping cluster NG 50 with a size of 1.02 Mb, and BUSCO analysis showed that up to 98.0% of complete genes could be detected in the assembly, resulting in a predicted 30,436 protein-coding genes [11]. At the same time, it was revealed for the first time that the NCED gene in the β -carotenoid metabolic extension pathway is the key gene to regulate the color formation of apricot pulp, which provides a strong reference for apricot gene identification and breeding strategy. Zhang et al. selected “silver fragrant white” apricot as sequencing material, and used three-generation sequencing technology, second-generation data error correction, and HiC sequencing for sequence assembly, obtaining a high-quality 8-apricot chromosome sequences with a genome size of 251.19 Mb, heterozygosity of 0.99%, and annotation of 29,230 protein coding genes. The repeat sequence in this variety genome was 46.78%, significantly higher than the 38.28% observed in the “Chuanzhi” apricot [12]. 

In this study, 53 common apricot varieties were collected from Shaanxi, Shanxi, Inner Mongolia, and Gansu. By applying WGRS technology and SNP as markers, comprehensive mining genetic variation information revealed the different sources of common apricot germplasm and genetic diversity, providing evidence for variety breeding and efficient use in the future.

## 2. Materials and Methods

### 2.1. Materials

A total of 53 common apricot varieties were obtained from breed bases in different regions of China, covering most of the typical Chinese apricot varieties. A total of 13 varieties from Hohhot of Inner Mongolia (HS), 8 from Jinzhong City of Shanxi Province (TG), 16 from Datong City of Shanxi Province (DT), 11 from Yulin City of Shaanxi Province (SX), and 5 from Lanzhou City of Gansu Province (LZ) were collected. Samples comprising 2 g of fresh, healthy, and pest-free young leaves were selected as test samples and sent to the sequencing company for genome sequencing.

### 2.2. DNA Extraction

Using the E.Z.N.A. Tissue DNA Kit, we first completed the digestion and lysis of tissue samples within 30 min, then added 500 μL of RB Buffer pre-mixed with 2-mercaptoethanol. The sample was purified using a gDNA Filter Column and a HiBind^®^ RNA Mini Column, which included centrifugation at 14,000× *g* for 5 min, transferring the clear lysate, adding 0.5 volumes of ethanol, centrifugation at 12,000× *g* for 1 min, repeating the sample transfer, and washing steps with 400 μL of RWF Wash Buffer and 500 μL of RNA Wash Buffer II, ultimately obtaining pure DNA (>3 µg; concentration > 30 ng/µL; OD 260/OD 280 = 1.80~2.00).

### 2.3. Library Preparation and Sequencing

To prepare for Illumina pair-end sequencing, we required at least 3 μg of genomic DNA per sample to create libraries with inserts around 450 bp. Following Illumina’s protocol, DNA was fragmented by Covaris, blunt-ended with T4 DNA polymerase, and ‘A’ bases were added to the 3′ ends for adapter ligation. After purifying the desired fragments by gel-electrophoresis, we enriched and amplified them with PCR, incorporating index tags. The libraries were then quality-checked and sequenced on the Illumina NovaSeq 6000 platform (150 bp*2 paired-end reads) by Shanghai Biozeron Biotechnology Co., Ltd. (Shanghai, China) .The filtered valid data were aligned with the reference genome (the published “Yinxiangbai” apricot genome, (https://www.ncbi.nlm.nih.gov/datasets/genome/GCA_020424065.1/ (accessed on 3 December 2024)) using BWA v0.7.12-r1039 software. Sequencing depth and coverage were calculated using custom Perl scripts, and SNPs and short InDels were detected via the “Haplotype Caller” function of GATK v4.1.2.0 software using a valid BAM file. The Variant Call Format (VCF) files were generated by quality filtering, and the SV of samples was detected using the software BreakDancer v1.1.2 to obtain high-quality SNPs for genetic diversity analysis. 

### 2.4. Data Analysis

Based on the filtered markers, kinship analysis was performed using GCTA v1.93.2 software to calculate genetic distances and obtain a G matrix (genetic relationship matrix) between two samples. The evolutionary tree was constructed by FastTree v2.1.10 software (neighbor-joining methods, model: p-distance). Both the eigenvalue (Eigen value) and the eigenvector (Eigen vector) were calculated to draw the PCA map. Population structure analysis was performed using Admixture v1.3.0 software, and the K values were taken as 2 to 10. The best K value was determined based on the crossover error rate.

## 3. Results

### 3.1. DNA and Sequencing Quality Control

DNA was extracted from the complete and pest-free leaves of healthy plants of 53 apricot varieties. The concentration ranged from 16.5 and 60.6 ng/μL, with an average concentration of 27.81 ng/μL. The total amount was between 1.65 and 6.06 μg, with an average value of 2.78 μg. After electrophoresis, there were no impurities such as protein or pigment, and qualified DNA was used for library sequencing. According to the quality control analysis of the sequence data, the Q20 value ranged from 97.08% to 98.52%, with a mean value of 98.04%; Q30 ranged from 91.78 to 95.63%, with a mean value of 93.81%; and the GC value was between 37.91% and 41.44%, with a mean value of 39.61%. All the above results indicate that the sequencing quality of this study was high and met the data requirements. Using the genome of “*Yinxiangbai*” apricot as the reference genome, the 53 samples had a sequencing depth between 7.31~80.01×, and an average sequencing depth of 17.29×. The maximum alignment value was 98.1%, with a minimum value of 95.87%, and a mean value of 97.66%, The average coverage was 90.50%. The above results are shown in Table 1, and the similarity of each sample to the reference genome met the criteria for subsequent analysis. 

### 3.2. Variant Analysis

**Figure 1:**
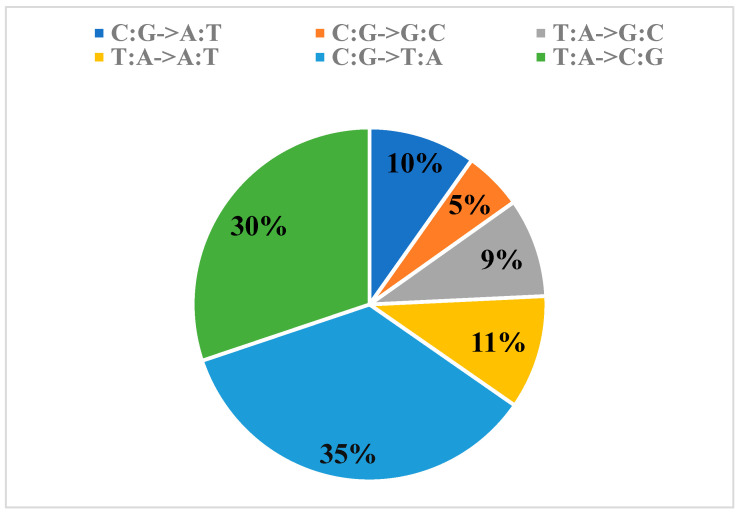
Statistics on the number of different types of single base substitutions.

A total of 88,332,238 SNP sites and 13,934,301 InDel sites were obtained by annotating the SNP and InDel sites obtained from variant detection using GATK. SNP point variation ranged from 939,061 to 3,548,841. Among the SNPs, 54.8% (48,392,511) were located in intergenic regions, 7.3% (6,436,463) were in exons, 16.8% (14,853,605) were intronic, 10.1% (8,897,323) were 1 Kb upstream of the gene, and 9.3% (8,246,616) were 1 Kb downstream of the gene (Table 2). Variations in the coding region gene may cause changes in traits by changing the amino acid sequence. Among them, 3,028,064 SNPs were synonymous mutations, meaning that base substitutions would not cause amino acid alterations. In contrast, 3,323,008 SNPs were non-synonymous mutations, indicating that base substitutions would lead to changes in coding amino acids. The range of InDel variation was observed to be between 149,830 and 402,481. The deletion variant occurred between 1379 and 4516, and the insertion variant occurred between 737 and 2743. Further details regarding InDel can be found in Table 3. The magnitude of selection pressure acting on genes can be assessed by calculating the ratio of nonsynonymous and synonymous mutations. Results greater than 1 indicate that the gene is under positive selection pressure, while a result of less than 1 indicates that the gene is under negative selection pressure. A result equal to 1 indicates that the gene is under neutral selection pressure [13]. The ratio of nonsynonymous mutations (3,323,008) to synonymous mutations (3,028,064) was 1.10, indicating that the gene is under positive selection pressure. SNP variant types were annotated (Figure 1), and 65% (85,795,736) of SNPs were base converted (ts), while 35% (45,528,853) were base switched (tv). The SNP variant types were dominated by base conversion. The base conversion/base subversion value (ts/tv) was 1.88, indicating that the SNP variant types were mostly dominated by variation between nucleotides of the same type, among which LNLJX had the highest number of variants (3,951,322) and the lowest ts/tv value (1.77), indicating that fewer gene exchange events occurred in it (Supplementary Files S1 and S2).

### 3.3. G Matrix Analysis

**Figure 2:**
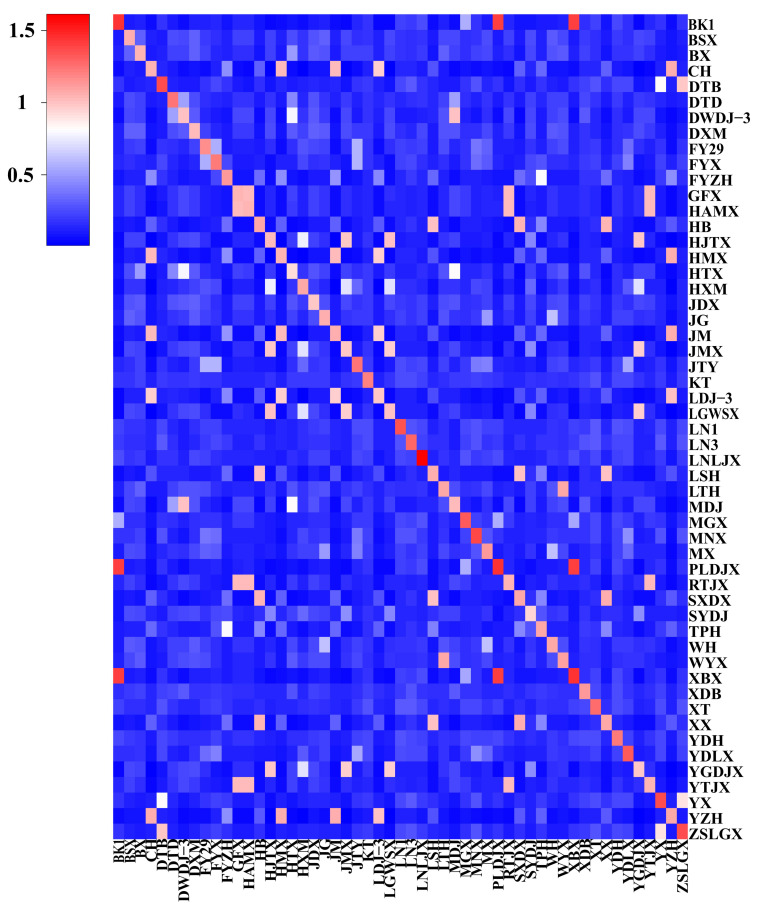
Heat map of sample genetic relationships. Pairs from the first sample to the last. The larger the value, the closer to red; that is, the closer the relationship between two individuals.

The genomic relatedness of the G matrix can be used to resolve the influence of factors involving unclear population genealogy or unclear ancestors on the population structure analysis [14]. Based on the selected markers, GCTA v1.93.2 software was used to obtain a binary G matrix (genetic relationship matrix). Larger G values indicate a closer relationship between breeds. A heatmap was drawn using the G matrix, resulting in Figure 2. A total of 1378 relatedness values were obtained from the relationship analysis (Supplementary File S3), ranging from 1.41 to 0.01, with an average of 0.18, in which *PLDJX* and *BK1* were the closest related (G = 1.41), and yzh and *LGWSX* had the most distant relationship (G = 0.01). 

### 3.4. Cluster Analysis

**Figure 3:**
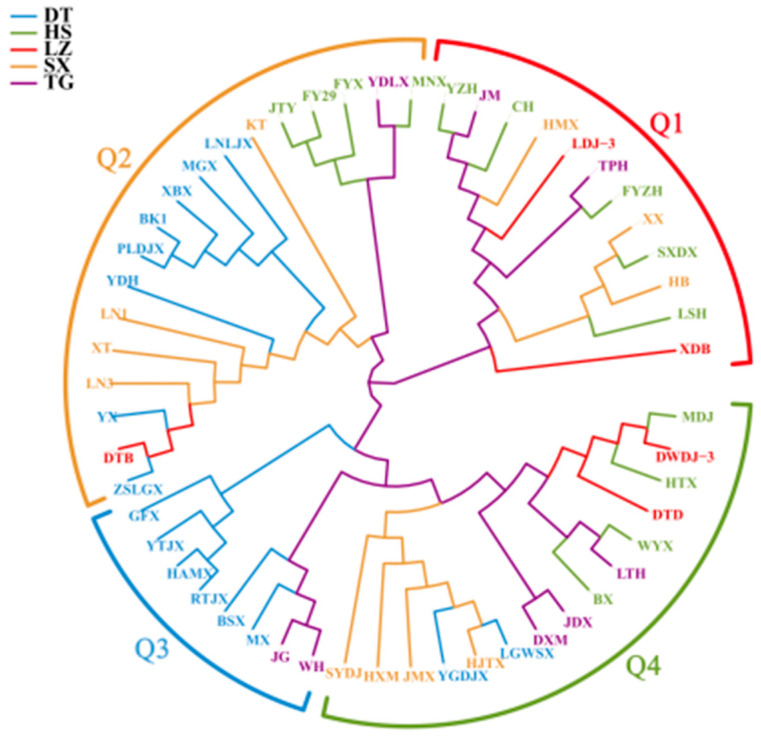
Comparison of genetic relationship matrices and genetic distances. DT: Sample from Datong City, Shanxi Province; HS: from Hohhot, Inner Mongolia; LZ: samples from Lanzhou City, Gansu Province; TG: from Jinzhong City, Shanxi Province; SX: from Yulin City, Shaanxi Province. A total of 53 common apricot varieties were classified into four taxa (Q1, Q2, Q3, and Q4).

The genetic distance was calculated by GCTA v1.93.2 software (Supplementary File S4). The genetic distance between the 53 common apricot varieties ranged between 0.00367 to 0.264344. Among them, the genetic distance between WYX and YX was the greatest (0.264344), and the genetic distance between HMX and JM was the closest (0.00367). The NJ phylogenetic tree was constructed using FastTree 2.1.10. The results are shown in Figure 3. Firstly, through cluster analysis, the 53 varieties were divided into four groups named Q1, Q2, Q3, and Q4, colored in red, orange, green, and blue, respectively. Q1 consisted of 12 varieties with a genetic distance range of 0.00367 to 0.23945. In total, five varieties were collected from HS, two from LZ, three from SX, and two from TG. The genetic distance between HMX and JM was the closest among all the varieties. Q2 was composed of 18 varieties, with a genetic distance range of 0.005383 to 0.252468. Eight varieties were collected from DT, four from HS, one from LZ, four from SX, and one from TG. Among the varieties, BK1 and XBX exhibited the closest genetic distance. Q3 was composed of 8 varieties, with observed genetic distances spanning a range from 0.004959 to 0.228316. Six varieties originated from DT; and Two from TG. RTJX and YTJX exhibited the closest genetic distance to each other. Q4 consisted of 15 varieties, and the genetic distance range was 0.004558 to 0.236309. Two varieties originated from DT; Four from HS; Two from LZ; four from SX; three from TG. 

### 3.5. Kinship Relationship Analysis

Both G matrices and phylogenetic trees can be used to infer population structure and relatedness among varieties [14,15]. By comparing the results of these two analysis methods, we can determine the real situation of kinship. 

The results of genetic relationship analysis based on G matrix and genetic distance showed that some individuals had a close genetic relationship. The top 30 G matrices with a close genetic relationship and the genetic relationship combination of cluster analysis are listed, respectively, in Table 4. It was found that 26 varieties appeared in both analyses, but the rankings were slightly different. 

### 3.6. Principal Component Analysis

**Figure 4:**
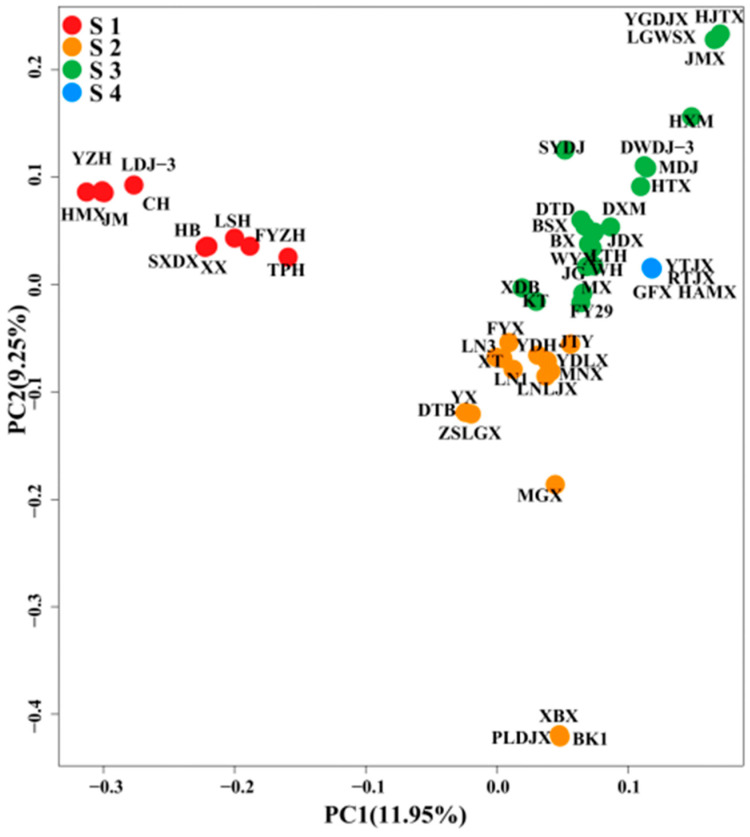
PCA, principal component analysis. Each color represents a group of species. S1 (Red): Group I; S2 (Orange): Group II; S3 (Green): Group III; S4 (Blue): Group IV.

The kinship of 53 common apricot varieties was analyzed by principal component analysis, and the evolution of different apricot varieties was classified. According to the PCA two-dimensional clustering diagram (Figure 4), PCA1 and PCA2 explained 11.95% and 9.25% of the total variance, respectively. The 53 varieties were divided into four groups: S1 (red), S2 (orange), S3 (green), and S4 (blue). S1 was discovered to be far away from the other three, so S1 is significantly different from S2, S3, and S4. The distance between S1, S2, and S3 is close, indicating that the similarity is high.

### 3.7. Population Genetic Structure Analysis

**Figure 5:**
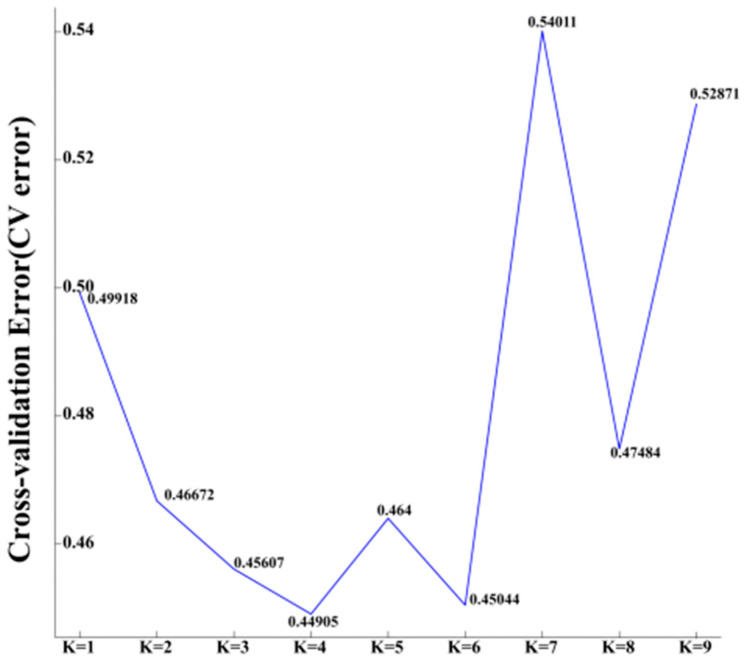
Cross-validation error rate line chart.

**Figure 6:**
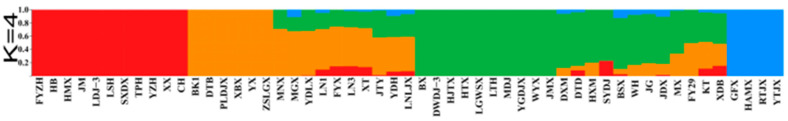
Analysis of SNP population structure in 53 common apricot varieties (K = 4). Each column of vertical grids represents the genetic background of a sample; each color block represents an estimated ancestor, and the proportion of the vertical grid occupied by each color block represents the proportion of that ancestor that contributes to the genetic background of that sample. Red for G1; Orange for G2; Green for G3; Blue for G4.

To further understand the evolutionary history and kinship of apricot, the required atlas files were generated using PLINK v1.9. Population genetic structure analysis of the 106,002,914 SNP loci obtained was performed using Admixture v1.3.0 software. The number of clusters (K value) was assumed to be 2 to 9, and clustering was then carried out. According to the cross-validation error rate (C ross-validation error, CV error), the optimal number of clusters was determined. The CV value with the smallest cross-validation error rate corresponded to the optimal number of clusters [16]. The results are shown in Figure 5. The CV value was the smallest when K = 4, indicating that the 53 common apricot samples were divided into four clusters that closely reflected the real situation of the group. As shown in Figure 6, the four clusters were named G1, G2, G3, and G4, and the main color segments were red, orange, green, and blue, respectively. The results are shown in Figure 6. There were 11 varieties in G1 (red) with pure genetic backgrounds, namely, *FYZH*, *HBX*, *HMX*, *JM*, *LZDJX*, *LSX*, *SXDJX*, *TPH*, *YZH*, *XX*, and *CH*. There were 16 varieties in G2 (orange), of which 6 varieties had pure genetic backgrounds: *BK1*, *DTB*, *PLDJX*, *XBX*, *YX*, and *ZSLGX*. The other varieties contained a variety of genetic components and were mixed. There were 22 varieties in G3 (green), of which 6 varieties had pure genetic backgrounds: *BX*, *DWDJ-3*, *HJTX*, *HTX*, *LGWSX*, *LTH*, *MDJ*, *YGDJX*, *WYX*, and *JMX*. Other varieties contained multiple genetic components and were hybrids. G4 (blue) had four varieties with pure genetic backgrounds, namely, *GFX*, *HAMX*, *RTJX*, and *YTJX*. 

**Figure 7:**
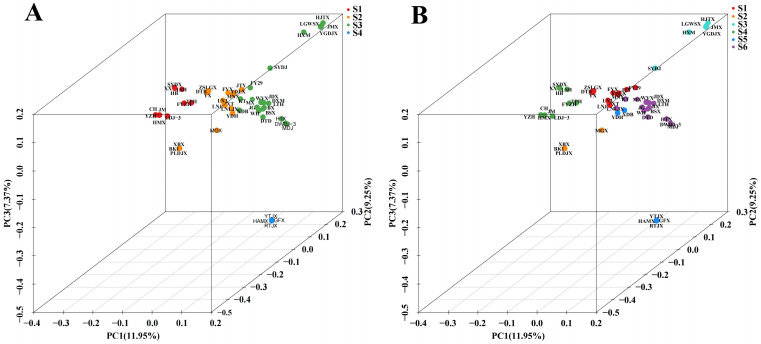
Three-dimensional PCA distribution maps of different groups. (**A**) The three-dimensional PCA of the samples when the varieties are divided into four groups; (**B**) the three-dimensional PCA of the samples when the varieties are divided into six groups. Varieties with the same color in the PCA plot were considered to be in the same line.

When the optimal number of clusters was determined according to the CV error, it was found that the CV value was also small when the 53 common apricot samples were divided into six lineage populations. Therefore, the PCA three-dimensional graph was used for reverse verification. As shown in Figure 7, when the samples were divided into six groups, it was more chaotic than when they were divided into four groups. Thus, the grouping result was optimal when the sample was divided into four lineage populations.

## 4. Discussion

The cultivation history of apricot in China can be traced back to 3000 years ago. Due to the frequent blind germplasm exchange among many local varieties, the genetic background of apricot varieties is unknown and their kinship relationships are less understood [5]. At the same time, due to the large number of varieties of germplasm resources varieties in breeding units, deviations in genealogy or variety name records are inevitable during introduction, propagation, and seed preservation, leading to results exhibiting different names for the same object, as well as identical names for different entities [17]. This has led to genetic variation, the determination of kinship, the establishment of core breeding groups, and the exploration of high-quality germplasm resources. Therefore, it is particularly important to collect, organize, and analyze the genetic diversity of common apricot varieties. 

Compared with other molecular marker techniques, SNP analysis is based on a single nucleotide. SNPs are widely used in the analysis of population genetic diversity due to their wide distribution, large number, and strong genetic stability. However, it has high cost, is prone to false positives, and has a high error rate [18]. Therefore, filtering standards need to be strictly controlled. In this study, 53 high-quality common apricot germplasm resources were selected, using the “*Yinxiangbai*” apricot genome as the reference genome. A total of 88,332,238 high-quality SNPs were identified, with an average sequencing depth of 17.29, a Q20 of 98.04%, a Q30 of 93.81%, and a GC value of 39.61%. The ratio of nonsynonymous to synonymous mutations was 1.10, which was greater than that of peach [19]. The ts/tv value of 1.89 indicated that the SNP variant types were mostly those between the same nucleotides, compared with wild tea trees [20] and bubble tung [21]. The research results were consistent. *LNLJX* had the highest number of variants and the lowest ts/tv values, indicating fewer gene exchange events. 

Our analysis was based on the construction method of genome relationships via the G matrix, proposed by Van Raden [22]. Through G matrix analysis, G values between 1.41 and 0.01 were found, and the relationship between *PLDJX* and *BK1* was determined close. Through clustering analysis of 53 apricot varieties divided into four groups, genetic distances between 0.264344 to 0.00367 were discovered, with an average of 0.225. With the exception of the fourth group, which had a relatively similar geographical location, varieties from the five geographical locations were distributed across the other three groups. *HMX* and *JM* were found to be closely related, followed by *CH* and *JM*, *JMX* and *YGDJX*. Comparing the results of the two analyses, although the order of affinities was different, both indicated that some of the individuals had closer affinities. 

The results of this study are similar to those obtained by Zhang et al. Common apricots were divided into four groups, and the results of PCA analysis and population genetic structure analysis were highly consistent with our analyses [7]. The analysis of phylogenetic trees was slightly different. In the individual evolutionary tree, *XDB* of Group 1 (Q1) and FY29 and KT of Group 2 (Q2) were divided into Group 3 (G3) for population genetic structure analysis, which may be due to the complex genetic structure of these three varieties.

From the perspective of geographical distribution, neither population structure analysis nor the phylogenetic tree classified geographically similar species into categories, which is consistent with the results of Gao et al. According to their research, it is possible that the genetic communication between apricot groups is too frequent [23]. 

## 5. Conclusions

In this study, 96,765,900 high-quality SNPs were obtained by whole-genome resequencing analysis of 53 common apricot varieties. These SNPS are not only useful for analyzing the variations in common apricots, but also help to study the species characteristics and population evolution of apricots and locate the gene loci of target traits. This enables us to quickly discover the genetic variations related to important traits in apricots, which can be applied in molecular breeding and can shorten the breeding cycle. The relationships and genetic structures for 53 common apricot varieties were also determined. This provides a theoretical basis for the development and identification of allelic varieties with phenotypic effects in the future.

## Figures and Tables

**Table 1:** Mapping ratio statistics.

Number	Sample Name	Sample Code	Alignment Rate (%)	Coverage (%)	Aver-Dep
1	Huangjintianxing	*HJTX*	97.57	90.04	9.25
2	Hongmeixing	*HMX*	97.96	89.77	15.48
3	Lingnong3	*LN3*	98.04	89.46	16.93
4	Lingnong1	*LN1*	97.95	89.07	15.55
5	Hongxiangmi	*HXM*	97.85	92.60	13.15
6	Huangbanxing	*HB*	97.72	90.05	15.36
7	Jinmeixing	*JMX*	97.79	90.82	15.58
8	Kaitexing	*KT*	98.00	88.99	12.52
9	Sanyuandajiexing	*SYDJ*	97.67	91.20	16.55
10	Xiangxing	*XX*	97.53	89.95	15.20
11	Xintexing	*XT*	97.68	89.76	15.16
12	Liaoninglijiexing	*LNLJX*	95.87	88.89	13.13
13	Hamixing	*HAMX*	98.10	89.46	14.84
14	Boke1	*BK1*	97.84	89.67	14.94
15	Muguaxing	*MGX*	97.92	90.03	16.94
16	Gongfoxing	*GFX*	97.43	89.91	13.92
17	Meixing	*MX*	97.64	90.19	16.22
18	Yingtiaojinxing	*YTJX*	97.89	89.46	14.77
19	Xiangbaixing	*XBX*	97.95	90.02	14.81
20	Yidianhong	*YDH*	97.70	89.39	13.92
21	Yanggaodajiexing	*YGDJX*	98.07	90.69	14.47
22	Pingliangdajiexing	*PLDJX*	97.79	89.67	14.00
23	Zaoshuliguangxing	*ZSLGX*	97.96	88.79	13.47
24	Ruantiaojinxing	*RTJX*	97.92	89.40	14.55
25	Liguangwanshuxing	*LGWSX*	97.97	89.94	13.14
26	Youxing	*YX*	97.53	89.95	15.20
27	Baishuixing	*BSX*	97.50	93.08	63.27
28	Jiguang	*JG*	97.76	92.55	36.41
29	Wanhong	*WH*	97.85	93.27	40.78
30	Jinmei	*JM*	97.47	93.27	80.01
31	Jidanxing	*JDX*	97.40	90.61	11.82
32	Yidalixing	*YDLX*	97.75	89.49	9.96
33	Daxingmei	*DXM*	97.55	90.59	12.22
34	Luotuohuang	*LTH*	97.81	89.82	9.97
35	Taipinghong	*TPH*	97.42	91.24	19.09
36	Laoshanhong	*LSH*	96.92	89.66	8.64
37	Mengdajiexing	*MDJ*	96.10	89.93	10.13
38	Wuyuexian	*WYX*	97.79	88.27	7.31
39	Shanxidajiexing	*SXDX*	97.43	89.29	10.14
40	Jintaiyang	*JTY*	97.61	92.70	10.66
41	Yanzhihong	*YZH*	97.63	88.30	7.52
42	Fenyanzhihong	*FYZH*	97.77	91.86	31.76
43	Baixing	*BX*	97.96	96.43	12.26
44	Manaixing	*MNX*	97.92	88.72	8.58
45	Houtouxing	*HTX*	97.79	94.84	12.68
46	Chuanhong	*CH*	97.83	90.05	11.86
47	Fengyuanxing	*FYX*	97.69	90.90	14.64
48	Fengyuan29	*FY29*	97.76	90.58	11.28
49	Xindianbaohexing	*XDB*	97.82	90.40	13.45
50	Lanzhoudajiexing	*LDJ-3*	97.65	91.43	13.80
51	Dongwudajiexing	*DWDJ-3*	97.32	90.87	12.10
52	Dongxiangdajiexing	*DTD*	97.76	89.10	11.89
53	Dongxiangbaohexing	*DTB*	97.48	89.90	14.67

**Table 2:** SNP annotation information table.

Genomic Position	SNP Number of Loci
Nonsynonymous	3,323,008
Stop gain	68,981
Synonymous	3,028,064
Stop loss	16,410
Exonic	6,436,463
Intronic	14,853,605
Intergenic	48,392,511
Upstream	8,897,323
Downstream	8,246,616
Upstream/Downstream	1,480,979
Splicing	24,741

**Table 3:** InDel annotation results table.

Genomic Position	InDel Number of Point Points
Frameshift deletion	170,474
Frameshift insertion	88,397
UTR5	381
Non-frameshift deletion	81,518
Non-frameshift insertion	57,623
Stop gain	8807
Stoploss	3344
Downstream	1,741,285
Exonic	410,163
Intergenic	7,513,932
Intronic	2,954,011
Splicing	12,788
Upstream	1,931,934
Upstream/Downstream	328,046

**Table 4:** Comparison of genetic relationship matrices and genetic distances.

Sample Combination	Genetic Distance	Genetic Distance Ranking	Affiliation	Relationship Ranking
*PLDJX × BK1*	1.4122059	1	0.005700	17
*PLDJX × XBX*	1.4097709	2	0.005570	16
*XBX × BK1*	1.4068449	3	0.005380	14
*LTH × WYX*	1.0724071	4	0.008011	26
*YZH × HMX*	1.0648852	5	0.006841	25
*JM × YZH*	1.0563859	6	0.005550	15
*JM × YZH*	1.0540115	7	0.006232	21
*CH × YZH*	1.0524688	8	0.006614	23
*HBX × SXDJX*	1.0507799	9	0.006166	19
*XX × HBX*	1.0439993	10	0.004860	4
*JM × HMX*	1.0262081	11	0.004150	1
*RTJX × HAMX*	1.0208997	12	0.005090	12
*YTJX × HAMX*	1.0196109	13	0.005020	9
*HAMX × CH*	1.0188594	14	0.004860	5
*RTJX × YTJX*	1.0188064	15	0.004960	7
*HAMX × GFX*	1.0187374	16	0.005050	11
*RTJX × GFX*	1.0187374	17	0.005030	10
*JM × CH*	1.017809	18	0.003750	2
*YTJX × GFX*	1.0164344	19	0.005000	8
*LZDJX × YZH*	1.010440 7	20	0.010713	30
*LGWSX × HJTX*	0.9968687	22	0.006652	24
*DWDJ-3 × MDJ*	0.9960544	24	0.006255	22
*HJTX × YGDJX*	0.9889232	26	0.006221	20
*HJTX × JMX*	0.9878738	27	0.006029	18
*YGDJX × LGWSX*	0.9703597	29	0.005100	13
*LGWSX × JMX*	0.9690487	30	0.004900	6

## Data Availability

All data are available upon reasonable request.

## Supplementary Materials

The following supporting information can be downloaded at: https://www.mdpi.com/article/10.3390/cimb46120844/s1, Supplementary Files S1–S4.
